# Gigwa v2—Extended and improved genotype investigator

**DOI:** 10.1093/gigascience/giz051

**Published:** 2019-05-11

**Authors:** Guilhem Sempéré, Adrien Pétel, Mathieu Rouard, Julien Frouin, Yann Hueber, Fabien De Bellis, Pierre Larmande

**Affiliations:** 1 Centre de coopération Internationale en Recherche Agronomique pour le Développement (CIRAD), UMR INTERTRYP, F-34398 Montpellier, France; 2 South Green Bioinformatics Platform, Bioversity, CIRAD, Institut National de la Recherche Agronomique (INRA), IRD, Montpellier, France; 3 INTERTRYP, Univ Montpellier, CIRAD, Institut de Recherche pour le Développpement (IRD), Montpellier, France; 4 DIADE, Univ Montpellier, IRD, 911 Avenue Agropolis, 34394 Montpellier, France; 5 Bioversity International, Parc Scientifique Agropolis II, 34397 Montpellier Cedex 5, France; 6 CIRAD, UMR AGAP, F-34398 Montpellier, France; 7 AGAP, Univ Montpellier, CIRAD, INRA, Institut national d'études supérieures agronomiques de Montpellier (Montpellier SupAgro), Montpellier, France

**Keywords:** genomic variations, VCF, HapMap, PLINK, NoSQL, MongoDB, SNP, indel, web, interoperability, REST, BrAPI, GA4GH

## Abstract

**Background:**

The study of genetic variations is the basis of many research domains in biology. From genome structure to population dynamics, many applications involve the use of genetic variants. The advent of next-generation sequencing technologies led to such a flood of data that the daily work of scientists is often more focused on data management than data analysis. This mass of genotyping data poses several computational challenges in terms of storage, search, sharing, analysis, and visualization. While existing tools try to solve these challenges, few of them offer a comprehensive and scalable solution.

**Results:**

Gigwa v2 is an easy-to-use, species-agnostic web application for managing and exploring high-density genotyping data. It can handle multiple databases and may be installed on a local computer or deployed as an online data portal. It supports various standard import and export formats, provides advanced filtering options, and offers means to visualize density charts or push selected data into various stand-alone or online tools. It implements 2 standard RESTful application programming interfaces, GA4GH, which is health-oriented, and BrAPI, which is breeding-oriented, thus offering wide possibilities of interaction with third-party applications. The project home page provides a list of live instances allowing users to test the system on public data (or reasonably sized user-provided data).

**Conclusions:**

This new version of Gigwa provides a more intuitive and more powerful way to explore large amounts of genotyping data by offering a scalable solution to search for genotype patterns, functional annotations, or more complex filtering. Furthermore, its user-friendliness and interoperability make it widely accessible to the life science community.

## Background

Nowadays, next-generation sequencing technologies have become a standard tool for many applications in basic biology as well as for medicine and agronomic research. With the decreasing cost of genome sequencing, many laboratories are increasingly adopting genotyping technologies as routine components in their workflows, generating large datasets of genotyping and genome sequence information. Additionally, scientists are also interested in re-using data produced by large international consortia that have performed re-sequencing or high-density genotyping on material from representative, publicly available diversity collections. For instance, the 3000 Rice Genome Project [1] and the 1000 Plants Project [2] provide huge amounts of sequence variation data to search and download through, respectively, their SNP-SEEK [3] or 1001genomes.org portals. Such information is not easy to handle because of its size and its complex structure, both unsupported by standard software such as spreadsheet processors. This kind of data is indeed mostly made available as variant call format (VCF) [4] and often needs to be converted into specific software formats for subsequent analyses (e.g., PLINK [5], Darwin [6], Flapjack [7]). In addition, the tools available to filter data or perform more complex operations are mainly available in the command line. Because these results may contain tens of millions of variants for thousands of samples, scalable and user-friendly solutions need to be offered to the biological community.

We thus developed Gigwa [8] with the aim of providing a system that helps relieve biologists from the burden of technical aspects of variation data manipulation. Gigwa is a web application designed to store large volumes of genotypes (up to tens of billions), initially imported from VCF or other file formats, in a NoSQL database (MongoDB [9]), and to provide a straightforward interface for filtering these data. It makes it possible to navigate within search results, to visualize them in different ways, and to re-export subsets of data into various common formats. In the first version published in 2016, we focused our work on the following important aspects: (i) filtering features that include genotype pattern search, e.g., minor allele frequency (MAF) and missing data ratio to name a few; (ii) storage performance by choosing a NoSQL engine and designing data structure in order to scale with growing dataset sizes and support incremental addition of data into projects; (iii) sharing capabilities, i.e., enabling multiple users to efficiently work on the same datasets without the need to replicate them; and (iv) graphical visualization, which allows either a summarized or detailed view of the dataset contents.

Our experience with biologists operating in various research fields and studying different species helped us improve the application with regard to many aspects. In version 2, we overhauled the graphical interface to improve user experience and visualization features. This new release also integrates a data and user management section to facilitate system administrators’ work. We took the evolution of next-generation sequencing and analysis software into account by adding new import and export formats. Gigwa's scaling capacities along with its speed performance were also improved, thus making it able to deal with much larger datasets. Finally, we enabled interoperability with other applications, in particular by implementing standard representational state transfer (REST) application programming interfaces (APIs).

Since the release of Gigwa version 1 [8], the application has been adopted by several institutes, in some cases embedded within information systems like the Musa Germplasm Information System [10], in others deployed as a self-sufficient portal providing convenient access to public data [11]. Feedback was thus collected, suggesting ideas for significant improvement. In this article, we describe the list of newly added features, provide details about software improvements, discuss the benchmarking work done to assess performance progress, and finally present a concrete use case showing the usefulness and efficiency of the application.

## Findings

### Newly added features

#### Administration interface

A fully featured administration interface has been implemented, allowing for managing databases, projects, users, and permissions. Thus, it is now possible to manage data visibility and sharing, to suppress existing data, and to grant users read or write permissions on datasets, all with a few mouse-clicks without the need to interact with configuration files as before.

#### New import functionalities

The first version of Gigwa only supported importing data via specification of an absolute path on the webserver's filesystem. While this method is still supported because it is useful to administrators, new ways of feeding genotyping data into the system have been added:

- By uploading files from the client computer (either using drag and drop or by browsing the filesystem);
- By providing http(s) URLs to online files;
- By specifying the base-URL of a BrAPI [12] v1.1 compliant service that supports genotyping data calls. Indeed, this version embeds a client implementation of BrAPI, which allows users to select a genome map and a study in order to feed a Gigwa project with corresponding genotypes pulled from the BrAPI datasource.

Additionally, the application now allows anonymous users to import genotyping data as temporary databases for filtering purposes. Such datasets are only guaranteed to be maintained online for a limited period. An adjustable size limit can be set for files uploaded by any users, including anonymous ones.

As for import formats, the PLINK (PLINK, RRID:SCR_001757) [5] flat-file standard format is now also supported as input for genotyping data.

Finally, version 2 supports enriching permanent databases by importing metadata as tabulated files for the individuals they refer to. Those metadata facilitate individual selection in the interface based on complementary information beyond the individual identifier (e.g., passport data, traits).

#### Supported annotation formats

The application is able to take into account functional annotations present in VCF files in order to allow end-users to filter on them. The first version was only able to parse annotations originating from SnpEff (SnpEff, RRID:SCR_005191) [13], whereas version 2 also supports annotations added by VEP (Variant Effect Predictor, RRID:SCR_007931) [14].

#### New export functionalities

The export features have also been extended as follows:

- The ability to refine the individual list at export time has been added. It is therefore possible to selectively export data relating to a chosen subset of individuals, independently from the one used for filtering variants;
- A new export format was added (.fjzip) for compatibility with the Flapjack [7] software;
- In the case of data files being exported to server, Gigwa v1 provided the means to push this output to a running instance of the IGV (Integrative Genomics Viewer, RRID:SCR_011793) [15] stand-alone software. Version 2 additionally supports pushing it to online tools such as Galaxy (Galaxy, RRID:SCR_006281) [16, 17] or SNiPlay [18]. The list of connected tools can be managed by administrators, and a custom tool can be configured by each end-user.

#### New filtering capabilities

Gigwa v2 introduces the following new filtering functionalities:

- In the case where individuals are numerous, defining group contents can be fastidious: selection can now be conveniently made by filtering individuals based on imported metadata. The selection made in each group can then be saved in the web browser using the localStorage API [19].
- For data imported from the VCF format, the initial version supported applying thresholds on the per-sample read depth (i.e., DP) and genotype quality (i.e., GQ) fields. The system now provides means to filter genotypes using any genotype-level numeric fields. The availability of such fields is automatically detected and corresponding threshold widgets are dynamically built into the interface when applicable.
- Two groups of individuals can now be defined for filtering. Therefore, any combination of genotype-level filters that was previously possible to express can now be applied to a first group, while a second combination of filters can be applied to a second group.
- One of the genotype patterns that could originally be applied to selected individuals was “All same,” resulting in selecting variants for which those individuals all had the same genotype. This option has been made more flexible (thus renamed to “All or mostly the same”) and may now be used in conjunction with a similarity ratio, i.e., a percentage defining how many of the selected individuals within the current group shall share the major genotype.
- Thanks to the 2 latter features, the system is now able to discriminate variants with regard to a phenotype. This may be achieved by defining groups according to the phenotype (e.g., resistant vs susceptible), choosing for both the “All or mostly the same” genotype pattern, setting a reasonable similarity ratio, and ticking the “Discriminate groups” checkbox that appears in this situation. This will result in selecting variants where most individuals in each group have the same genotype, that genotype being different between both groups. The usefulness of this functionality is illustrated in the “Gigwa in action” section.

#### Additional visualization functionalities

On top of the density graph, additional series can now be displayed representing any VCF-defined genotype-level numeric field. The underlying data for these series consist of the given field's cumulated values for a customizable selection of individuals. Thanks to this feature, the density of variants may now be observed with regard to numeric metadata fields such as genotype quality or read depth distribution.

#### APIs and data interoperability

Much effort has been put into making Gigwa data interoperable:

External, online genome browsers can now be configured for viewing each variant in its genomic context. Administrators have the ability to specify the URL of a default genome browser (e.g., GBrowse [GBrowse, RRID:SCR_006829] [20], JBrowse [JBrowse, RRID:SCR_001004] [21]) per database. End-users may override this default configuration by specifying another tool, thus only affecting their own interface. When such a configuration exists for a database, each variant line in the main browsing interface table features a clickable icon leading to opening the genome browser at the position of the variant so that it can be checked against available tracks.

Moreover, 2 REST APIs have been implemented to automatically provide access to any data imported into the system:

- The GA4GH [22] v0.6.0a5 API. The new graphical user interface (GUI) mentioned above is implemented as a client for this API; i.e., most interaction between Gigwa's client-side and server-side code is performed in compliance with the standards defined by the GA4GH API;
- The BrAPI [12] v1.1 API. Flapjack [7] and BeegMac [23] are examples of clients that are compatible with the data Gigwa serves via BrAPI. The Musa Germplasm Information System [10] also interacts with Gigwa through BrAPI by serving Gigwa-hosted data using a proxy approach.

Both APIs have different purposes and, respectively, work with health-related data and crop-breeding data. One clear overlap between them being the support for sharing genotyping data, we thought it relevant to implement for each API the calls that rely on the type of data held in our system.

#### Application architecture outline

**Figure 1:**
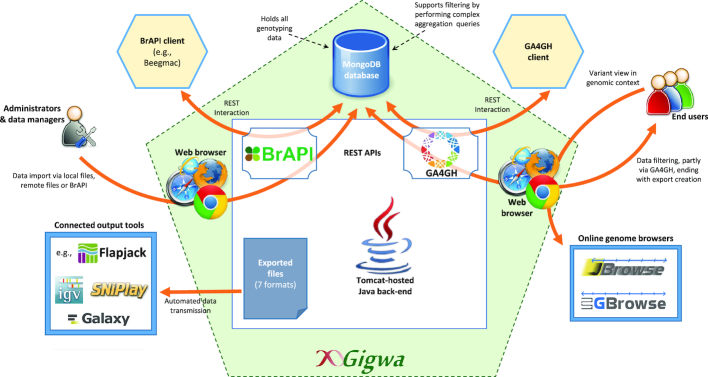
High-level diagram of Gigwa architecture and features.

Fig. 1 illustrates the architecture of Gigwa version 2 and summarizes its functionalities.

### Software improvements

#### Description

(i) User-friendly interface.

The entire web interface has been reworked and is now based on Bootstrap V3 [24], which makes it more self-consistent, intuitive, attractive, and cross-browser compatible. In addition, the new GUI brings various enhancements such as support for decimal numbers for filters applying to numeric values, and the aforementioned facilities for selecting individuals (cf. “New filtering capabilities” section).

Additionally, a web page was added to the interface to allow users to watch process progress when importing genotyping data, or exporting them to a physical file on the webserver (direct downloads require the web browser to remain open at all times and therefore cannot benefit from this feature). Each progress-watching page has a unique URL and can thus be re-opened at any time. This feature is particularly convenient when working with large amounts of data because of the time taken by imports and exports.

(ii) Enhanced performance in terms of query speed.

As a reminder, each search operation is performed via multiple MongoDB aggregation queries targeting evenly sized variant chunks, thus improving response times while allowing progress monitoring.

The data storage structure has also been tuned to optimize speed performance. Gigwa queries consist of combinations of filters that can be split into 2 categories:

- “variant-level” filters (variant type, number of known alleles, sequence and position) applying to indexed fields;
- “genotype-level” filters (all others) applying to non-indexed fields (mainly because a MongoDB collection cannot have >64 indexes, which would be sufficient with only few genotyped individuals).

In version 1, as described by Sempéré et al. [25], indexed fields were held in the "variants" collection only, while unindexed fields (mainly genotype-related) were held in the "variantRunData" collection. Thus, any query involving both types of filters resulted in the following scenario:

- Create a temporary variant collection (subset of the "variants" collection) based on a variant-level query;
- Use the latter collection to restrict genotype-level query target to the variants that matched the variant-level query;
- Update the temporary collection's contents to keep only the variants also matching the genotype-level query.

Although this method worked satisfactorily, it did not scale efficiently with dataset size. Indeed, in the case of a lenient variant-level query, the system would spend much time writing into the temporary collection (copying most of "variants"’ contents) and also updating it afterwards (especially when the genotype-level query was stringent).

In version 2, all searchable contents in the variants collection are duplicated into variantRunData, thus allowing all filters to be applied simultaneously by querying a single collection. This data duplication is small and leads to a negligible volume increase that is advantageously compensated by other structure modifications (e.g., removal of empty genotype fields for missing data). This improvement is illustrated in Additional File 1.

In addition, the use of temporary collections has been reduced to a minimum. Previously, any filtering resulted in the creation of temporary data. Thus, only the browsing and exporting of an entire (i.e., unfiltered) database were performed on the main variants collection. With version 2, a temporary collection is created only if a genotype-level query has been submitted. Thus, when the query applies solely to variant-level fields, it is remembered and re-applied to the main variants collection when browsing or exporting data. These indexed queries being extremely fast to execute, responsiveness is not affected even on very large datasets.

Additionally, the JavaScript Object Notation syntax of search queries has been optimized to reduce both the number of operations involved in applying filters and the amount of data processed at each stage of MongoDB's aggregation framework.

Finally, a “multithreading regulation” mechanism was implemented, which adjusts the number of concurrent threads at run time when executing queries. It is based on the database server's live responsiveness and therefore automatically adapts to the current load without taking hardware considerations into account. More detail can be found in Additional File 2.

(iii) Enhanced filtering workflow, improving responsiveness.

When the search button is clicked, depending on the status of the “Enable browse and export” checkbox, the system either builds—and keeps track of—the list of matching variants (find procedure) or simply returns a count value telling how many matching variants were found (count procedure). Each query count is cached as an array of sub-values (1 for each genome chunk), the sum of which equals the query's total result count. These cached values are used when the same query is invoked anytime later; they allow instant response for the count method, and faster response for the find method (thanks to MongoDB's $limit operator, which prevents the aggregation pipeline engine from searching further than the last matching variant in each chunk). In version 1, the count method was always executed prior to the find method, thus almost doubling unnecessarily the execution time when the box was checked. In this situation, version 2 overcomes this problem via a find method that supports a “count at the same time” option. This way, the query is only executed once with a negligible overhead, resulting in much faster display of the results and access to export functionalities.

(iv) Enhanced export and visualization features.

When creating export files, instead of synchronously reading data chunks from the database and writing them to the output stream, we implemented 2 separate processes, one dedicated to reading, the other dedicated to writing, both designed to run concurrently. The reading process was optimized using a multithreading regulation routine as described above.

As for the density visualization functionality, it was improved by making chart zooming dynamic: a new query is now sent to the server each time the zoom level changes, thus always ensuring optimal data resolution.

#### Benchmarking

We performed benchmarking tests to (i) assess how tools tested in our previous article evolved in terms of speed, (ii) demonstrate the benefit of targeting a genome region when applying a genotype-level filter in Gigwa, and (iii) evaluate our system's capacity to work with very large datasets.

Two hardware configurations were used in this benchmark:

Configuration 1: comparable to the one tested in the original benchmark [8], and essentially used for assessing the progress made since then. It is a Hewlett Packard EliteBook 850 G3 laptop computer with an Intel Core i7–6500U central processing unit (CPU) at 2.50 GHz, 16 GB of random access memory (RAM), and a Samsung PM871 512 GB (6Gbit/s) TLC SSD 850.

Configuration 2: high-performance machine typically suitable to serve as a production environment for MongoDB and thus for Gigwa. We used it to evaluate the performance of the latest software versions running on production hardware, including on large datasets. It is a Dell PowerEdge R640 server based on an Intel Xeon Gold 5122 CPU at 3.60 GHz, 384 GB of RAM, and a 1.92To SAS (12Gbit/s) Toshiba PX05SV SSD.

Two datasets were used in this benchmark:

Dataset 1: dataset tested in the original benchmark, the Old Subset SNP Dataset v0.2.1 (formerly named CoreSNP v2.1) from the 3000 Rice Genomes Project [26], containing genotypes for 3,000 individuals on 365,710 single-nucleotide polymorphisms (SNPs). Its reasonable size (4.4 GB in VCF format) was suitable for experimenting with Configuration 1.

Dataset 2: filtered SNP v1.0 Dataset from the 3000 Rice Genomes Project, containing genotypes for 3,024 individuals on 4,817,964 SNPs (VCF file of 60.4 GB, preliminarily annotated with SnpEff v4.3T).

Because it was demonstrated in the original article that relational database management system−based implementations were suitable only for querying on indexed fields (which Gigwa v1 could do nearly as efficiently) but not on genotype-level information, such solutions were left out in the present work. Therefore, we mostly concentrated on executing queries at the genotype level, especially using the MAF range query, which is among the most CPU-intensive. All Gigwa instances were set up with MongoDB's WiredTiger storage engine, using the zlib compression level, which had seemed to be the best option in the original tests.

**Figure 2:**
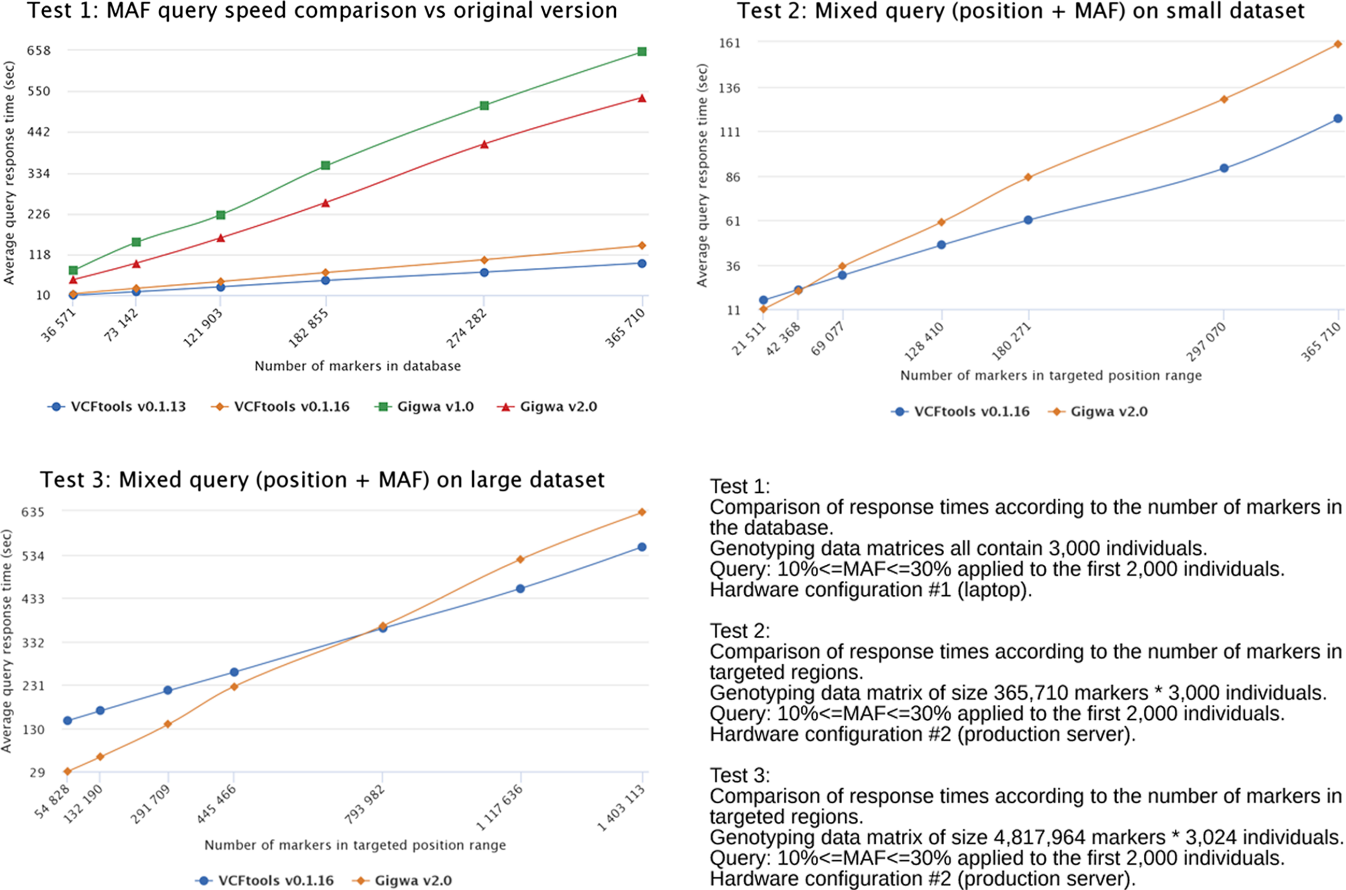
Benchmark results.

Three different comparison tests were run in this benchmark, which are described in Table 1 and whose results are reported in Fig. 2

**Table 1.** Benchmarking test description

Test No.	Aims	Methods
**1**	Assess evolution of tool speed performance. Involved Gigwa v1, Gigwa v2, VCFtools v0.1.13 (originally benchmarked) [4], and VCFtools v0.1.16 (latest at assessment time)	Run on configuration 1 using dataset 1 (along with sub-sampled versions, so as to obtain 6 different databases), all with the same number of individuals (i.e., 3,000) but with various numbers of markers. Query was a MAF range between 10% and 30% applied to the first 2,000 individuals
**2**	(i) Assess performance of latest versions of tools (Gigwa v2 and VCFtools v0.1.16) when simultaneously querying on variant-level (indexed in Gigwa) and genotype-level (unindexed in Gigwa) fields. (ii) Estimate the benefit of migrating to high-performance hardware by monitoring differences in response times between tools	Run on configuration 2 using dataset 1 without its derivatives, sub-sampling now being performed on the fly by restricting the search to a varying list of chromosomes. The query was the same MAF range query as above
**3**	(i) Test Gigwa v2’s suitability for working on very large datasets. (ii) Compare trends with those observed in a small dataset (Test 2)	Run on configuration 2 using dataset 2, sub-sampling being performed on the fly by restricting the search to a varying list of chromosomes. The query was the same MAF range query as above

Average response times were calculated based on the results provided in Additional File 3.

Looking at Test 1 trends, and considering the results of the original benchmark, the speed difference between VCFtools (VCFtools, RRID:SCR_001235) and Gigwa increased substantially. Because the binaries used in both tests were the same for the versions initially assessed, this increase is due to hardware matters (the amount of RAM was reduced from 32 to 16GB). The difference observed stems from the fact that Gigwa, being a 3-tier web application, cannot be as lightweight as VCFtools and thus requires more memory to achieve similar performance (cf. Test 2).

The main goal in this test was to compare results tool by tool, thus assessing speed evolution between former and current versions. A substantial speed gain ranging between 18.5% and 36.5% was observed in moving from Gigwa v1 to Gigwa v2. However, rather oddly, a consistent speed loss ranging between 40% and 48.5% was observed in moving from VCFtools v0.1.13 to v0.1.16.

From Test 2 results, a first observation is that using production hardware in which much RAM is available for MongoDB and Tomcat, the difference in speed between tools is far smaller. If we take the full dataset (365,710 variants) as a comparison reference, the Gigwa v2 query takes 3.8 times longer to execute than with VCFtools v0.1.16 for Test 1, whereas for Test 2 it is only 1.36 times slower.

Besides, when targeting a region of the genome, Gigwa takes advantage of its indexing strategy and even responds faster than VCFtools as the given region becomes narrow enough.

Test 3 results demonstrate that Gigwa v2 is able to efficiently handle and search very large datasets (here, >14 billion genotypes) when running on suitable hardware. Also, the trend observed in Test 2 is confirmed here, i.e., targeting a precise genome region for applying a genotype-oriented filter is of great benefit in terms of speed.

##### Benchmark discussion

The benchmarking work previously performed by Sempéré et al. [8] had shown that, and provided reasons why VCFtools excels in executing genotype-level queries on an entire large dataset. Although equaling its performance in a 3-tier application like the one presented here does not seem feasible for such queries, we thought it relevant to assess the progress made since version 1, still in comparison with VCFtools. This work led to several conclusions: (i) Gigwa v2 performs substantially better than v1 in applying genotype-level queries; (ii) setting up Gigwa on production hardware (with large amounts of RAM) greatly improves its performance; (iii) combining variant-level and genotype-level filters whenever possible is a good way to make the most of Gigwa's indexed fields and can lead it to outperform VCFtools.

In a separate work lying outside the scope of this article, we tested Gigwa v2 configured as a sharded cluster on a single server (Configuration 2). We observed a speed gain within the 20–30% range, which we consider interesting, but we acknowledge the complexity that it induces in terms of application deployment and maintenance. Further investigation would therefore be required to propose best practices in deploying an optimized configuration.

### Gigwa in action

In order to demonstrate the user-friendliness of the application, we selected a research study that reported the identification of a major quantitative trait locus (QTL) for sex determination in *Pundamilia* (a genus of cichlid fish), which was achieved by construction of a linkage map [27]. Because the genotype and phenotype files had been made available by the authors [28], it was straightforward to load them into Gigwa, assign all males (144) to group 1 and all females (78) to group 2, and apply a discrimination filter between them, with missing data maximum set to 10% and similarity ratio set to 90% for both groups. By ticking the discrimination filter, we made sure to restrict the results to variants showing a difference between groups.

**Figure 3:**
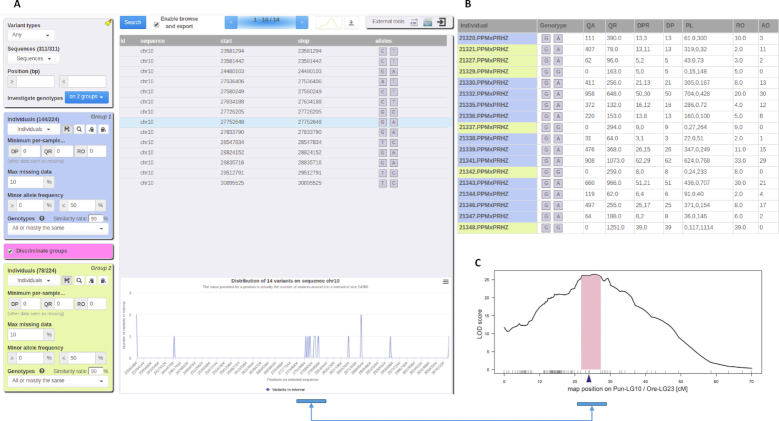
Discriminating variants. A, Filtering parameters and variant distribution. B, A discriminated variant's genotype with complementary information (males in blue, females in yellow). C, Chromosome region as reported by Feulner et al. [27] showing the strongest association with sex determination.

As shown in Fig. 3, 14 matching variants were found outright on the sole chromosome 10, all but 4 of them concentrating in the 27.53–29.52 megabase region. Findings from the original study indeed indicate that Pun-LG10 “acts as an (evolving) sex chromosome,” and that “the QTL region (Bayesian confidence interval) for sex determination in *Pundamilia* is located between 27.8 and 29.7 Mb” (Fig. 3C). Interestingly, by fine-tuning the similarity ratio as a cursor, we noticed that increasing it to 92% narrowed down results to variants exclusively concentrated in the mentioned QTL, while decreasing it to 89% revealed a few variants on unanchored scaffolds that could potentially be interpreted as belonging to Pun-LG10. Besides, we spotted the 2 individuals, 21321 and 21327 (Fig. 3B), that were labeled as females but had a male genotype as mentioned by Feulner et al. [27]. This shows that Gigwa can support rapid data exploration in order to provide a valuable indication for similar research studies. Through this example, we demonstrate that our software, although clearly not a replacement for methods such as genome-wide association studies or QTL mapping, provides a means to quickly obtain rough trends regarding the relationships between phenotypes and loci or genotypes, with only a few clicks.

## Conclusions

Gigwa v2 is a user-friendly, species-agnostic web application for managing and exploring high-density genotyping data. The software can be installed on a local computer or deployed as a data portal. It supports various standard import and export formats and provides advanced filtering options as well as means to visualize density charts or push selected data into various stand-alone or online tools. It implements 2 standard REST APIs: GA4GH, which is health-oriented, and BrAPI, which is breeding-oriented, thus offering wide possibilities of interaction with other systems.

Once installed, which is done by simply decompressing a zip archive for “stand-alone” users, its management interface obviates the need for any particular computer skills for users to administer, publish, or share their data.

Since its original version, Gigwa's data structure and query syntax have been optimized to a point where its speed performance is comparable to that of state-of-the-art command-line tools when running on production hardware. For instance, this version is able to deal with datasets as large as the 3000 Rice Genomes CoreSNP (genotypes for 3,024 individuals on 4,817,964 SNPs). Current live instances listed at [29] provide access to a range of diverse public datasets [26, 30–33] as well as video demonstrations to facilitate use and adoption.

Gigwa v2 allows for anonymous users to import their own data into temporary databases, thus allowing anyone to test the system on the mentioned live instances, for a limited duration. Its filtering functionalities are advanced enough to rapidly obtain an overview of variants discriminating 2 groups of individuals.

The type of data managed by this application being central to many kinds of studies in the genomics field, a wide range of extensions can be envisioned in terms of metadata support, downstream analyses, or visualization. In addition, speed improvements can still be envisioned by means of deep investigation of sharded cluster deployment possibilities.

## Availability of supporting source code and requirements

- Project name: Gigwa v2
- Project home page: http://www.southgreen.fr/content/gigwa
- Research Resource Identifier: Gigwa, RRID:SCR_017080
- Operating system(s): Platform-independent
- Programming languages: Java, MongoDB, HTML, Javascript
- Requirements: Java 8 or higher, Tomcat 8 or higher, MongoDB 3.4 or higher
- License: GNU Affero General Public License v3.0
- Restrictions to use for non-academics: None

## Availability of supporting data and materials

Gigwa's source code is available in the South Green GitHub repository [34, 35]. Supplementary data, benchmarking material, and installation archives can be found in the *GigaScience* GigaDB repository [36].

## Additional files

Additional File 1: Improvement on execution of mixed queries.

Additional File 2: Multithreading regulation explained.

Additional File 3: Detailed benchmark figures.

## Abbreviations

API: application programming interface; CPU: central processing unit; GUI: graphical user interface; MAF: minor allele frequency; QTL: quantitative trait locus; RAM: random access memory; REST: representational state transfer; SNP: single-nucleotide polymorphism; VCF: variant call format.

## Competing interests

The authors declare that they have no competing interests.

## Authors’ contributions

A.P. implemented the GA4GH service and integrated it into the client-server communication code. A.P. and G.S. designed the new GUI. G.S. implemented all other improvements and additions, optimized the data structure and application speed, and designed and ran the benchmarks. J.F., Y.H., M.R., and F.d.B. helped debugging by deeply testing the system, and suggested new features. G.S., P.L., and M.R. wrote the manuscript. All authors read and approved the final manuscript.

## Funding

This work was performed in the frame of the I-SITE MUSE "AdaptGrass project", publicly funded through ANR (the French National Research Agency) under the "Investissements d'avenir" programme with the reference ANR-16-IDEX-0006.

## Supplementary Material

GIGA-D-18-00476_Original_Submission.pdf

GIGA-D-18-00476_Revision_1.pdf

GIGA-D-18-00476_Revision_2.pdf

Response_to_Reviewer_Comments_Original_Submission.pdf

Response_to_Reviewer_Comments_Revision_1.pdf

Reviewer_1_Report_Original_Submission -- Weihua Chen 12/16/2018 Reviewed

Reviewer_1_Report_Revision_1 -- Weihua Chen 3/6/2019 Reviewed

Reviewer_2_Report_Original_Submission -- Mitchell Machiela 12/27/2018 Reviewed

Reviewer_2_Report_Revision_1 -- Mitchell Machiela 3/20/2019 Reviewed

Supplemental Files
