# Supplementary material for: Gigwa v2—Extended and improved genotype investigator
Source: Gigascience. 2019 May 11;8(5):giz051. doi: 10.1093/gigascience/giz051 (PMC6511067; doi:10.1093/gigascience/giz051)
Supplement: Supplemental Files [file giz051_supplemental_files.zip › Additional file 1.pptx]

## Slide 1
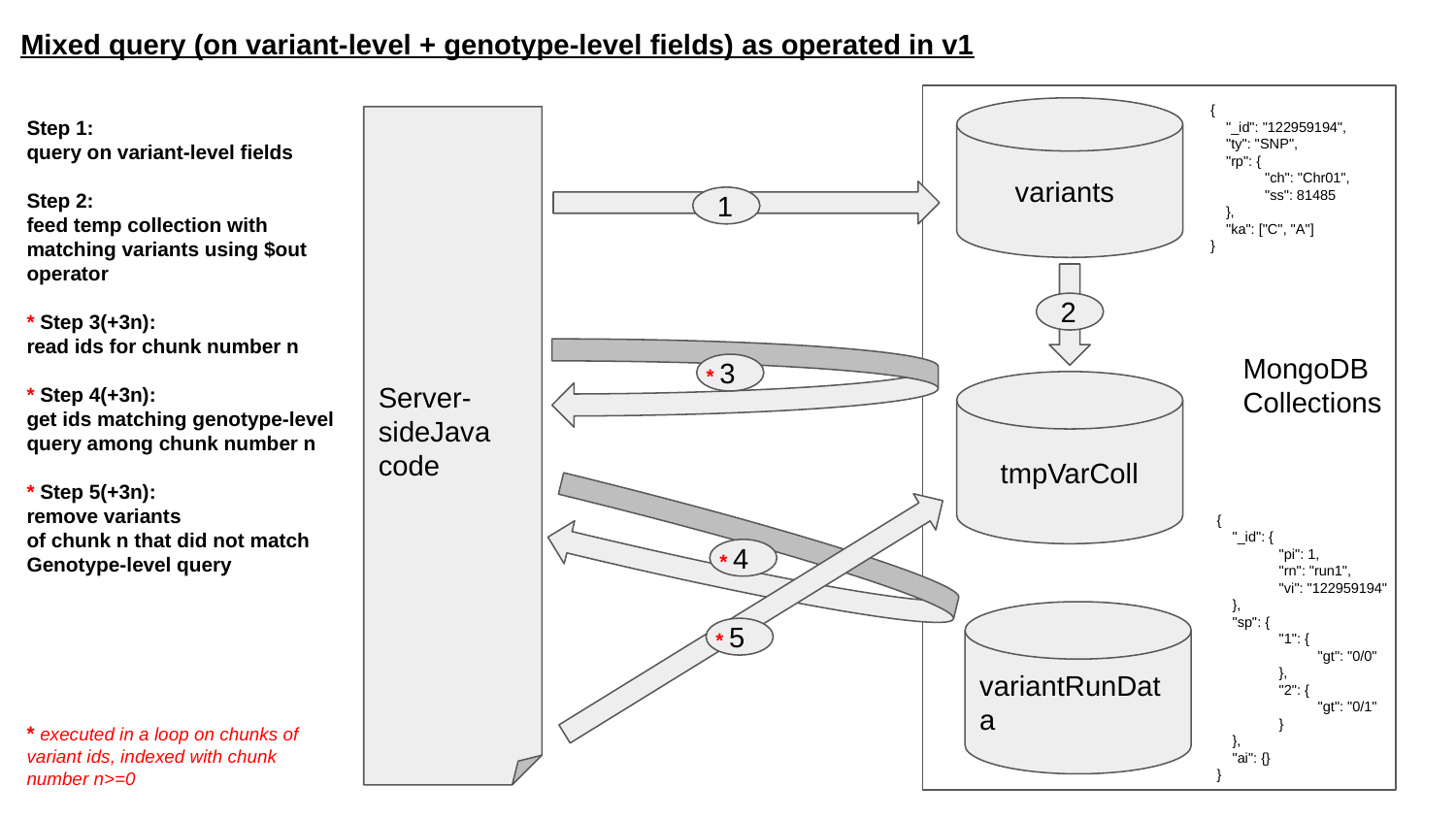

Mixed query (on variant-level + genotype-level fields) as operated in v1
MongoDB
Collections
{
 "_id": "122959194",
 "ty": "SNP",
 "rp": {
 "ch": "Chr01",
 "ss": 81485
 },
 "ka": ["C", "A"]
}
variants
Server-sideJava code
Step 1:
query on variant-level fields
Step 2:
feed temp collection with
matching variants using $out operator
* Step 3(+3n):
read ids for chunk number n
* Step 4(+3n):
get ids matching genotype-level query among chunk number n
* Step 5(+3n):
remove variants
of chunk n that did not match
Genotype-level query
* executed in a loop on chunks of variant ids, indexed with chunk number n>=0
1
2
* 3
tmpVarColl
{
 "_id": {
 "pi": 1,
 "rn": "run1",
 "vi": "122959194"
 },
 "sp": {
 "1": {
 "gt": "0/0"
 },
 "2": {
 "gt": "0/1"
 }
 },
 "ai": {}
}
* 4
variantRunData
* 5

## Slide 2
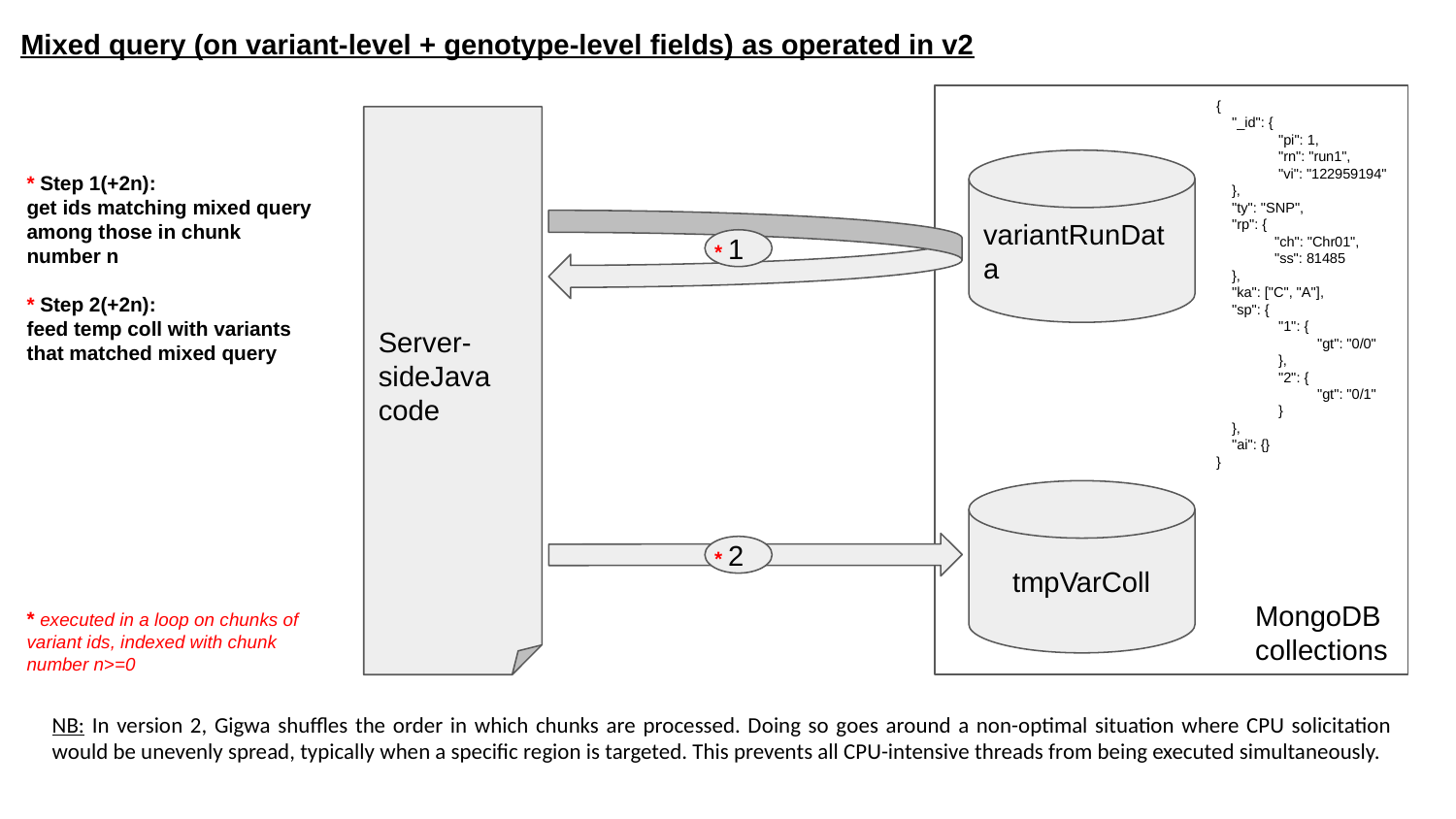

Mixed query (on variant-level + genotype-level fields) as operated in v2
{
 "_id": {
 "pi": 1,
 "rn": "run1",
 "vi": "122959194"
 },
 "ty": "SNP",
 "rp": {
 "ch": "Chr01",
 "ss": 81485
 },
 "ka": ["C", "A"],
 "sp": {
 "1": {
 "gt": "0/0"
 },
 "2": {
 "gt": "0/1"
 }
 },
 "ai": {}
}
MongoDB
collections
Server-sideJava code
variantRunData
* Step 1(+2n):
get ids matching mixed query among those in chunk number n
* Step 2(+2n):
feed temp coll with variants that matched mixed query
* executed in a loop on chunks of variant ids, indexed with chunk number n>=0
* 1
tmpVarColl
* 2
NB: In version 2, Gigwa shuffles the order in which chunks are processed. Doing so goes around a non-optimal situation where CPU solicitation would be unevenly spread, typically when a specific region is targeted. This prevents all CPU-intensive threads from being executed simultaneously.
