# Supplementary material for: Gigwa v2—Extended and improved genotype investigator
Source: Gigascience. 2019 May 11;8(5):giz051. doi: 10.1093/gigascience/giz051 (PMC6511067; doi:10.1093/gigascience/giz051)
Supplement: Supplemental Files [file giz051_supplemental_files.zip › Additional file 2.docx]

Multithreading regulation in Gigwa v2

In Gigwa version 2, a mechanism was implemented as an attempt to optimize the level of multithreading applied when running queries. As a reminder, each search operation is performed via multiple MongoDB aggregation queries targeting evenly sized variant chunks, thus allowing progress monitoring and improving speed performance. In the first version of Gigwa, the number of queries running simultaneously was constant and set to 5. With the aim of adapting to hardware performance, we found it relevant to monitor this number of simultaneous threads and readjust it on the fly while the search operation is running. This relies on 3 constant numbers: the minimum (m), maximum (M) and initial (i) numbers of simultaneous threads, respectively defined as 5, 50 and 10. If we call c the current number of simultaneous threads, then prior to launching the search process c is set equal to i. Threads are instantiated in a loop (with n being the index of the current one) and launched asynchronously, unless n is a multiple of c, in which case the thread is launched synchronously. Upon its completion, instead of simply waiting for all c threads to have terminated, as version 1 used to do, version 2 checks how many threads are still running among the c – 1 asynchronous ones. If this number is greater than c/2 then c gets divided by 1.5. If it is less than c/4 then c gets multiplied by 1.5. Otherwise it remains unchanged. The resulting c value is finally bound with M and m as upper and lower limits. This rule was adopted empirically after testing on various systems. This improvement is based on the server’s live responsiveness and therefore automatically adapts to the current load without taking hardware considerations into account. Its usefulness is illustrated in the benchmarking section below.

The flowchart below illustrates this mechanism.


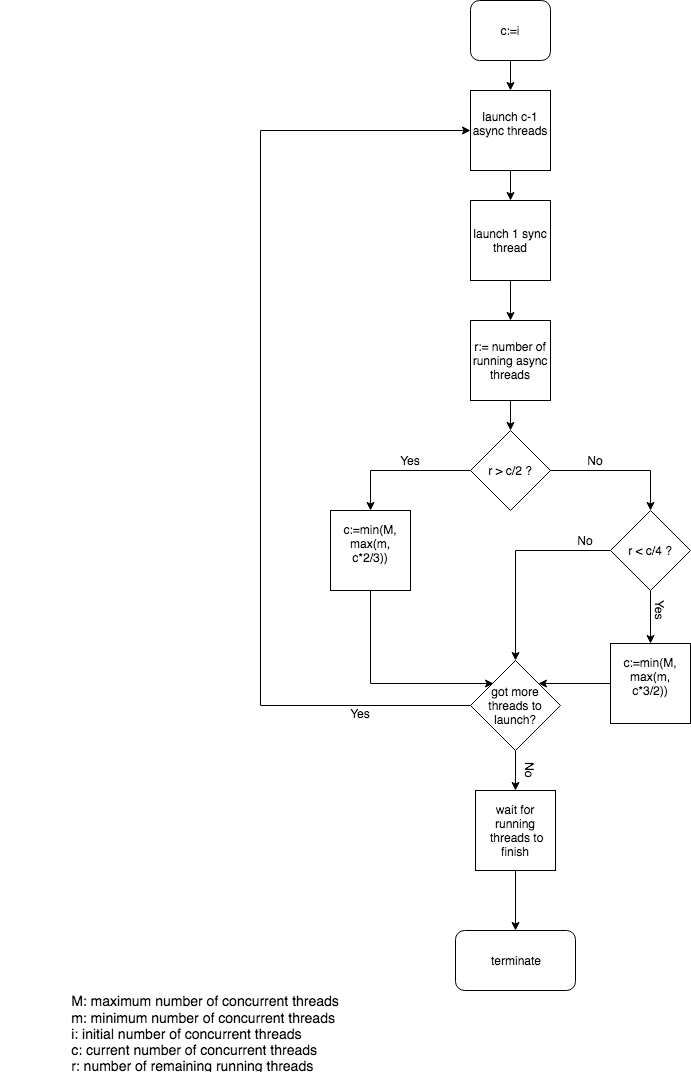


Benchmark assessing efficiency of this mechanism

Comparison of response times according to the number of filtered individuals: the number of targeted markers remained constant (i.e., those on chromosome 1, 576146) while the number of individuals was made variable.

Run on hardware configuration #2 (production server) using dataset 2 (genotypes for 3024 individuals on 4,817,964 SNPs)

Query: “Not all same” (filters out any variants for which all individuals have identical genotypes) among markers on chromosome 1.

Hardware configuration #2 (production server).


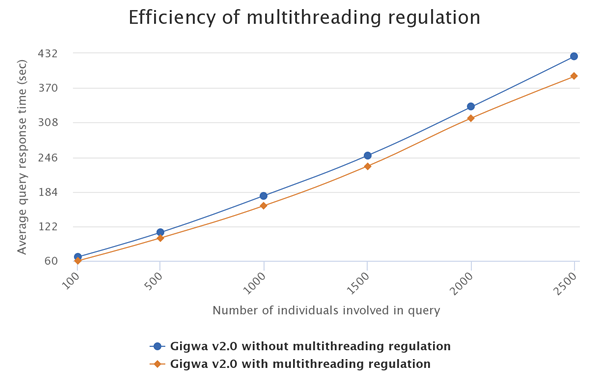


Figures lead to conclude that on this hardware configuration, regulating the number of concurrent threads improves speed by 9% on average.

Benchmark: detailed response times

| **NUMBER OF INDIVIDUALS** | **100** | **500** | **1000** | **1500** | **2000** | **2500** |
| --- | --- | --- | --- | --- | --- | --- |
|  |  |  |  |  |  |  |
| **AVERAGE RESPONSE TIME**  **without thread count regulation (sec)** | **67** | **111** | **177** | **249** | **336** | **427** |
|  | 65 | 111 | 177 | 250 | 334 | 425 |
|  | 72 | 112 | 175 | 250 | 338 | 434 |
|  | 65 | 109 | 178 | 247 | 337 | 423 |
|  |  |  |  |  |  |  |
| **AVERAGE RESPONSE TIME**  **with thread count regulation (sec)** | **60** | **101** | **159** | **230** | **316** | **391** |
|  | 62 | 111 | 151 | 231 | 306 | 394 |
|  | 57 | 101 | 162 | 229 | 312 | 387 |
|  | 61 | 92 | 164 | 230 | 331 | 392 |
|  |  |  |  |  |  |  |
| **FILTER RESULT** | **514262** | **569821** | **576088** | **576144** | **576146** | **576146** |
