# Supplementary material for: Gigwa v2—Extended and improved genotype investigator
Source: Gigascience. 2019 May 11;8(5):giz051. doi: 10.1093/gigascience/giz051 (PMC6511067; doi:10.1093/gigascience/giz051)
Supplement: Supplemental Files [file giz051_supplemental_files.zip › Additional file 3.docx]

**Test 1**

| **DATASETS** | **36571 MARKERS** | **73142 MARKERS** | **121903 MARKERS** | **182855 MARKERS** | **274282 MARKERS** | **365710 MARKERS** |
| --- | --- | --- | --- | --- | --- | --- |
|  |  |  |  |  |  |  |
| **AVERAGE RESPONSE TIME**  **for VCFTOOLS v0.1.13 (sec)** | **10** | **19** | **32** | **49** | **71** | **95** |
|  | 9 | 19 | 32 | 49 | 71 | 91 |
|  | 10 | 20 | 34 | 51 | 72 | 101 |
|  | 12 | 18 | 30 | 46 | 70 | 92 |
|  |  |  |  |  |  |  |
| **AVERAGE RESPONSE TIME**  **for VCFTOOLS v0.1.16 (sec)** | **14** | **28** | **46** | **70** | **104** | **141** |
|  | 14 | 29 | 47 | 70 | 104 | 147 |
|  | 13 | 27 | 45 | 73 | 102 | 138 |
|  | 14 | 27 | 46 | 68 | 106 | 138 |
|  |  |  |  |  |  |  |
| **AVERAGE RESPONSE TIME**  **for Gigwa v1 - zlib (sec)** | **75** | **150** | **223** | **353** | **513** | **655** |
|  | 75 | 149 | 223 | 355 | 514 | 656 |
|  | 78 | 160 | 256 | 355 | 505 | 635 |
|  | 70 | 139 | 224 | 352 | 514 | 660 |
|  | 71 | 150 | 262 | 348 | 514 | 658 |
|  | 80 | 151 | 252 | 357 | 517 | 668 |
|  |  |  |  |  |  |  |
| **AVERAGE RESPONSE TIME**  **for Gigwa v2 - zlib (sec)** | **51** | **95** | **162** | **256** | **411** | **534** |
|  | 54 | 97 | 160 | 252 | 411 | 569 |
|  | 53 | 91 | 171 | 243 | 425 | 521 |
|  | 50 | 94 | 143 | 246 | 451 | 513 |
|  | 43 | 94 | 162 | 276 | 404 | 515 |
|  | 53 | 100 | 172 | 264 | 366 | 561 |
|  |  |  |  |  |  |  |
| **FILTER RESULT** | **7007** | **14149** | **23489** | **35388** | **53259** | **70905** |

**Test 2**

| **NUMBER OF CHROMOSOMES** | **1 (#5)** | **1 (#1)** | **2 (#2, 11)** | **4 (#2, 6, 7, 11)** | **6 (#1, 3, 5, 8, 10, 12)** | **9 (#1, 2, 3, 4, 7, 9, 10, 11, 12)** | **ALL 12** |
| --- | --- | --- | --- | --- | --- | --- | --- |
| **NUMBER OF TARGETED MARKERS** | **21511** | **42368** | **69077** | **128410** | **180271** | **297070** | **365710** |
|  |  |  |  |  |  |  |  |
| **AVERAGE RESPONSE TIME**  **for VCFTOOLS v0.1.16 (sec)** | **16** | **22** | **30** | **47** | **61** | **90** | **118** |
|  | 16 | 22 | 30 | 47 | 61 | 90 | 113 |
|  | 16 | 22 | 30 | 47 | 61 | 90 | 120 |
|  | 16 | 22 | 30 | 47 | 61 | 90 | 120 |
|  |  |  |  |  |  |  |  |
| **AVERAGE RESPONSE TIME**  **for Gigwa v2 - zlib (sec)** | **11** | **21** | **35** | **60** | **85** | **129** | **160** |
|  | 11 | 22 | 35 | 57 | 82 | 137 | 154 |
|  | 11 | 22 | 33 | 64 | 86 | 127 | 163 |
|  | 11 | 20 | 37 | 60 | 87 | 123 | 164 |
|  |  |  |  |  |  |  |  |
| **FILTER RESULT** | **3422** | **8884** | **12935** | **22713** | **36889** | **55117** | **70905** |

**Test 3**

| **RANGE SIZE** | **First 5 Mbp in Chr6** | **First 10 Mbp in Chr6** | **First 20 Mbp in Chr6** | **31.25 Mbp (all Chr6 variants)** | **56.55 Mbp (Chr 11&12 variants)** | **79.75 Mbp (Chr 10, 11&12 variants)** | **102.69 Mbp (Chr 9, 10, 11&12 variants)** |
| --- | --- | --- | --- | --- | --- | --- | --- |
| **NUMBER OF TARGETED MARKERS** | **54828** | **132190** | **291709** | **445466** | **793982** | **1117636** | **1403113** |
|  |  |  |  |  |  |  |  |
| **AVERAGE RESPONSE TIME** **for VCFTOOLS v0.1.16 (sec)** | **147** | **170** | **217** | **260** | **362** | **455** | **552** |
|  | 148 | 171 | 217 | 260 | 361 | 455 | 553 |
|  | 147 | 170 | 219 | 260 | 363 | 456 | 551 |
|  | 146 | 169 | 215 | 260 | 362 | 455 | 553 |
|  |  |  |  |  |  |  |  |
| **AVERAGE RESPONSE TIME** **for Gigwa v2 - zlib (sec)** | **29** | **63** | **139** | **227** | **368** | **523** | **632** |
|  | 30 | 63 | 141 | 236 | 375 | 519 | 623 |
|  | 29 | 63 | 138 | 202 | 359 | 549 | 639 |
|  | 29 | 62 | 139 | 244 | 370 | 501 | 633 |
| **FILTER RESULT** | **13809** | **31725** | **57493** | **95365** | **223066** | **335710** | **406954** |
