# Supplementary material for: Gigwa v2—Extended and improved genotype investigator
Source: Gigascience. 2019 May 11;8(5):giz051. doi: 10.1093/gigascience/giz051 (PMC6511067; doi:10.1093/gigascience/giz051)
Supplement: Response_to_Reviewer_Comments_Original_Submission.pdf [file giz051_response_to_reviewer_comments_original_submission.pdf]

Author's Response To Reviewer Comments

Close

Dear editor and reviewers,

We followed your advice as thoroughly as possible. You will find below detailed answers to all remarks you made. The manuscript was slightly modified consequently.

In the hope that this revision meets the expected standards for publication in Gigascience, we remain at your disposal for any clarification that may be necessary.

Best regards,

Guilhem Sempéré

Reviewer #1

We are grateful for sharing your experience with us.

A PDF documentation was available on the project homepage, but probably not so easy to find. We took the following steps to improve documentation visibility and help users understand how to use Gigwa:

- we converted the existing documentation to HTML, enriched it by adding further explanation and inserting screenshots where it seemed relevant, and embedded it within the web application so that it can be opened at any time from the main menu.

- we created 4 demonstration videos with embedded comments showing how to import data, filter data, manage outputs and efficiently use the phenotype-based discrimination feature.

- we mentioned the availability of those videos at the top of the HTML documentation where we also provided a link to the homepage.

- we also improved the Swagger documentation page by providing the full relative path to mentioned calls and adding a table providing terminology correspondence between the internal data model and those of the REST APIs.

Since the review, Gigwa V2 was presented at the International Plant and Animal Genome conference (PAG), and aroused interest of scientists from various countries and institutes. Since then we are having feedback from people installing it, so we are confident that the number of users is going to grow significantly.

Regarding the number of citations, we are aware of this concern and therefore we applied the following changes to V2:

- we added a reference to the original publication on the web application's homepage

- we made the system include a HOW_TO_CITE.txt file in each exported archive

We hope that all these improvements will help our tool gain visibility in the near future.

Reviewer #2

(1) Thank you for this suggestion. We removed the 3 occurrences of the word "ergonomic" and replaced them with "easy to use", "straightforward" and "user-friendly".

(2) We added the following sentence to the "Description" paragraph of the Abstract: "The project home page http://www.southgreen.fr/content/gigwa provides a list of live instances allowing to test the system on public data (or reasonably-sized user-provided data)."

(3) The Gigwa V1 manuscript explains this in the following terms: "VCFtools excels here because it is a C++ program working on flat files, which means that the time needed to access each record is negligible, whereas database engines need to obtain/deflate objects before manipulating them". An additional reason is that Gigwa being a 3-tier application, the application server (Java code) is at all time exchanging data with either the client-side UI (Javascript code) or the database server (MongoDB). Reducing this data exchange to a minimum is one of the main aspects on which we focused when optimizing our code. In other words, not being as fast as highly specific command-line tools is the downside of providing a web interface on top of structured data. In order to make this clearer to readers, we split apart Benchmark results and Benchmark discussion. In the latter, we referenced the explanation made in the previous article. We also mentioned in that paragraph a separate investigation focused on estimating the benefit of deploying MongoDB as a sharded cluster.

(4) The live Gigwa instance mostly put forward is the one hosted by CIRAD, on the southgreen.fr domain. Since South Green, our platform and network of bioinformaticians, focuses on tropical and Mediterranean crops, including human data there would not have made much sense. However, the instance hosted by the IRD institution (http://gigwa.ird.fr/) is more suitable for this kind of data as some teams there work on human health. So, we listed all live instances hosted by our network on the application's homepage, mentioned that in the abstract, and imported the Hapmap project's phase2+3's data into the IRD instance. This dataset was annotated using snpEff, linked to the UCSC genome browser, and enriched with individual's metadata to help users make the best of it.

Close
