# Supplementary material for: Gigwa v2—Extended and improved genotype investigator
Source: Gigascience. 2019 May 11;8(5):giz051. doi: 10.1093/gigascience/giz051 (PMC6511067; doi:10.1093/gigascience/giz051)
Supplement: Response_to_Reviewer_Comments_Revision_1.pdf [file giz051_response_to_reviewer_comments_revision_1.pdf]

Author's Response To Reviewer Comments

Close

Dear editor and reviewers,

We are sorry that the IRD server had some downtime precisely when you tried to browse the human data we had put online for you. Please be sure that this issue was not at all related to our software itself but only to network configurations modified by system administrators in the process of server migrations.

The IRD server is now back online but unfortunately will go down again for about 24h on april 5th, which is the date at which all IRD servers will be physically moved to a different room. In order to provide you with an alternate solution we created a mirror of this Hapmap dataset on our CIRAD staging server, which is accessible at http://gigwa-dev.southgreen.fr/gigwaV2/?module=hg18_hapmap, and will remain so until the manuscript is officially accepted.

We re-uploaded the manuscript file with only two modifications :

- an update of reference number 12 (recently accepted paper)
- the insertion of the RRID reference in the Availability and requirements section

We were provided with an FTP access to your servers and are currently in the process of uploading benchmarking material.

Thanks again for your feedback and suggestions. Best regards,

Guilhem Sempéré

Close
