# Supplementary material for: Gigwa v2—Extended and improved genotype investigator
Source: Gigascience. 2019 May 11;8(5):giz051. doi: 10.1093/gigascience/giz051 (PMC6511067; doi:10.1093/gigascience/giz051)
Supplement: Reviewer_1_Report_Original_Submission -- Weihua Chen — 12/16/2018 Reviewed [file giz051_reviewer_1_report_original_submission.pdf]

Reviewer Report

Title: Gigwa v2 - Extended and improved genotype investigator

Version: Original Submission **Date:** 12/16/2018

Reviewer name: Weihua Chen

Reviewer Comments to Author:

In this manuscript, Sempere and colleagues presented Gigwa v2, an update of their previous version of the genotype investigator for genome wide analyses. During my brief test of Gigwa v2, I found that it was easy to use, did everything smoothly, and could everything they authors promised; the interface was smooth, informative and intuitive.
In principle, I would have no reason to turn down such a good tool. However, it worries me that the previous version got only six citations (or four according to ISI) since its publication in 2016. Although citations do not necessarily reflect the quality of a work, for a tool, it often means we got the audiences wrong. We made similar mistakes in the past in our lab, that is, we made a few tools that we thought would be useful to experimental biologists. However, very often, experimental biologists often found our tools very difficult to use; at the meantime, bioinformaticians were also reluctant to use them because they were more comfortable with command-line tools.
Here are some tips (that helped us greatly in the past) to attract audiences (experimental biologists in this case if I understand it correctly) :
1. to provide very detailed documentations for the tool, and
2. provide annotated screenshots whenever possible.

Level of Interest

Please indicate how interesting you found the manuscript: Choose an item.

Quality of Written English

Please indicate the quality of language in the manuscript: Choose an item.

Declaration of Competing Interests

Please complete a declaration of competing interests, considering the following questions:

- Have you in the past five years received reimbursements, fees, funding, or salary from an organisation that may in any way gain or lose financially from the publication of this manuscript, either now or in the future?
- Do you hold any stocks or shares in an organisation that may in any way gain or lose financially from the publication of this manuscript, either now or in the future?

- Do you hold or are you currently applying for any patents relating to the content of the manuscript?
- Have you received reimbursements, fees, funding, or salary from an organization that holds or has applied for patents relating to the content of the manuscript?
- Do you have any other financial competing interests?
- Do you have any non-financial competing interests in relation to this paper?

If you can answer no to all of the above, write 'I declare that I have no competing interests' below. If your reply is yes to any, please give details below.

I declare that I have no competing interests.

I agree to the open peer review policy of the journal. I understand that my name will be included on my report to the authors and, if the manuscript is accepted for publication, my named report including any attachments I upload will be posted on the website along with the authors' responses. I agree for my report to be made available under an Open Access Creative Commons CC-BY license (http://creativecommons.org/licenses/by/4.0/). I understand that any comments which I do not wish to be included in my named report can be included as confidential comments to the editors, which will not be published.

I agree to the open peer review policy of the journal

To further support our reviewers, we have joined with Publons, where you can gain additional credit to further highlight your hard work (see: https://publons.com/journal/530/gigascience). On publication of this paper, your review will be automatically added to Publons, you can then choose whether or not to claim your Publons credit. I understand this statement.

Yes Choose an item.
