# Supplementary material for: Gigwa v2—Extended and improved genotype investigator
Source: Gigascience. 2019 May 11;8(5):giz051. doi: 10.1093/gigascience/giz051 (PMC6511067; doi:10.1093/gigascience/giz051)
Supplement: Reviewer_2_Report_Original_Submission -- Mitchell Machiela — 12/27/2018 Reviewed [file giz051_reviewer_2_report_original_submission.pdf]

Reviewer Report

Title: Gigwa v2 - Extended and improved genotype investigator

Version: Original Submission **Date:** 12/27/2018

Reviewer name: Mitchell Machiela

Reviewer Comments to Author:

The authors present an improved version of their Gigwa tool, highlight feature improvements and perform some performance tests and use cases. The authors demonstrate this is a substantial improvement on their prior version. The paper is clear and well-written, although could be more concise and focused on the major changes rather than listing all changes made. I have a few minor suggestions to improve the overall manuscript and tool:
(1) I suggest removing the term "ergonomic" from the abstract and conclusion as this word does not capture well what the authors are attempting to communicate.
(2) A webaddress to the tool in the abstract would be useful for readers to quickly access the tool rather than having to read down to the Conclusions to find the site.
(3) I was expecting Gigwa to perform better than VCFtools, but this seems to only be the case for large datasets. Can the authors add more discussion on why VCFtools outperforms Gigwa? Are there other opportunities for Gigwa performance improvements?
(4) The Gigwa site does not include any data on human populations. There are several reference human genome datasets that would be useful to add to the site (e.g., HapMap, 1000 Genomes).

Level of Interest

Please indicate how interesting you found the manuscript: Choose an item.

Quality of Written English

Please indicate the quality of language in the manuscript: Choose an item.

Declaration of Competing Interests

Please complete a declaration of competing interests, considering the following questions:

- Have you in the past five years received reimbursements, fees, funding, or salary from an organisation that may in any way gain or lose financially from the publication of this manuscript, either now or in the future?
- Do you hold any stocks or shares in an organisation that may in any way gain or lose financially from the publication of this manuscript, either now or in the future?
- Do you hold or are you currently applying for any patents relating to the content of the manuscript?

- Have you received reimbursements, fees, funding, or salary from an organization that holds or has applied for patents relating to the content of the manuscript?
- Do you have any other financial competing interests?
- Do you have any non-financial competing interests in relation to this paper?

If you can answer no to all of the above, write 'I declare that I have no competing interests' below. If your reply is yes to any, please give details below.

I delcare that I have no competing interests

I agree to the open peer review policy of the journal. I understand that my name will be included on my report to the authors and, if the manuscript is accepted for publication, my named report including any attachments I upload will be posted on the website along with the authors' responses. I agree for my report to be made available under an Open Access Creative Commons CC-BY license (http://creativecommons.org/licenses/by/4.0/). I understand that any comments which I do not wish to be included in my named report can be included as confidential comments to the editors, which will not be published.

I agree to the open peer review policy of the journal

To further support our reviewers, we have joined with Publons, where you can gain additional credit to further highlight your hard work (see: https://publons.com/journal/530/gigascience). On publication of this paper, your review will be automatically added to Publons, you can then choose whether or not to claim your Publons credit. I understand this statement.

Yes Choose an item.
